# Sangre capilar, más allá de una historia de dinosaurios y unicornios

**DOI:** 10.1515/almed-2022-0110

**Published:** 2022-12-15

**Authors:** Álvaro González, Julia Maroto-García, Nerea Varo

**Affiliations:** Departamento de Bioquímica, Clínica Universidad de Navarra, Pamplona, España; Instituto de Investigación Sanitaria de Navarra (IdiSNA), Pamplona, España

**Keywords:** sangre capilar, preanalítica, telemedicina

Los especialistas más veteranos probablemente se acuerden del tiempo de hemorragia o tiempo de Ivy, una prueba propuesta a principios del siglo pasado para valorar la hemostasia primaria, y que, actualmente, duerme en los viejos libros tan antigua y fósil como los dinosaurios. Hoy en día, la sangre capilar seca es ampliamente conocida y usada en el laboratorio clínico, fundamentalmente, en los cribados neonatales, estudios farmacocinéticos, toxicológicos e infecciosos. Mediante una punción relativamente indolora en el talón o en el dedo de la mano se recogen unas gotas de sangre, unos pocos microlitros, en unos papeles de filtro especiales. Esta facilidad de toma de muestra y simplicidad de envío posterior la hace muy adecuada para programas de cribado de enfermedades neonatales. Así, la Organización Mundial de la Salud ha recomendado el uso de sangre seca en el cribado de infecciones, como la hepatitis B, C y el virus de la inmunodeficiencia humana (HIV) en países con infraestructura sanitaria deficitaria [1]. Sin embargo, en el laboratorio central, donde se procesan la gran mayoría de las muestras de sangre, prácticamente todas líquidas, su presencia es anecdótica.

Recientemente, hemos conocido la sentencia del juicio por fraude de la directora ejecutiva (CEO) de Theranos, una compañía que prometía, usando una tecnología propia, realizar los análisis empleando una gota de sangre capilar [2]. La historia de esta compañía, considerada inicialmente un unicornio en Silicon Valley, y su protagonista, una emprendedora que se llegó a comparar con Steve Jobs, tiene todos los ingredientes de una novela de intriga. Sin duda nos puede enseñar a inversores y laboratorios, lecciones de prudencia ante promesas no avaladas científicamente. Sin embargo, también hemos de reflexionar que esta compañía apuntaba a una de las necesidades más importantes de la sanidad, esto es, poder obtener muestras de sangre sin que el paciente acuda al centro hospitalario y realizar análisis con cantidad mínima de sangre.

La obtención de sangre capilar es un proceso relativamente indoloro, que puede realizar el propio paciente y en el que se pueden obtener pequeños volúmenes de sangre. Existen ya algunas compañías en diversos países, incluido España, que ofrecen perfiles analíticos bioquímicos en sangre capilar proporcionando el material para la obtención y envío de la muestra. La obtención remota de muestras de sangre sería muy útil a personas con acceso limitado a centros de extracción y también podría complementar el trabajo de los clínicos en el uso de la telemedicina. La pandemia ha hecho que muchos pacientes se hayan vuelto reacios a visitar centros hospitalarios. Estos centros se encuentran saturados en muchas ocasiones y la extracción remota liberaría de carga de trabajo al Servicio de Extracciones. Existen grupos de pacientes que se podrían beneficiar claramente del uso de pequeños volúmenes de sangre, como pueden ser niños, ancianos o enfermos crónicos y oncológicos que tienen extracciones frecuentes. También existen personas con miedo al propio proceso de extracción venosa para los que otro menos agresivo podría ser de mejor aceptación. No obstante, su obtención puede no ser recomendable en ciertos tipos de pacientes, como es en aquellos que tengan problemas circulatorios, inflamación en la zona de incisión o problemas de coagulación.

Y a esas necesidades debemos de atender los profesionales del laboratorio con una mirada científica y rigurosa. Un aspecto crítico se sitúa en la fase preanalítica que, aparentemente, sería la más sencilla, pero ha de ser realizada adecuadamente para conseguir un proceso robusto con una variabilidad aceptable. Las guías metodológicas, como la CLSI GP42-ED7 de 2020 [3], han de ser adaptadas para que el proceso lo pueda realizar una persona inexperta como es el paciente. Existen diversos dispositivos en el mercado diseñados para que el paciente pueda obtener pequeños volúmenes de sangre capilar por sí solo, desde lancetas a sistemas más sofisticados como los comercializados por Yourbio Health^®^ o Tasso^®^. Estos últimos dispositivos están diseñados para obtener muestra suficiente y evitar posible heterogeneidad, sobre todo, por contaminación con líquido intersticial o intracelular que se podría producir en el “ordeñado” del dedo. También una manipulación inadecuada puede causar una hemólisis de la muestra. Existen en el mercado tubos de hasta 1 mL para la recogida de estas muestras capilares, similares a los tubos pediátricos de uso común, y se podrían usar directamente en los autoanalizadores del laboratorio. Dado que las muestras se obtienen fuera del laboratorio y pueden tardar más de un día en llegar es importante disponer de un sistema de transporte que asegure la trazabilidad de la muestra y la estabilidad del analito a medir. El transporte, realizado habitualmente por medio de compañías logísticas, ha de cumplir la legislación en cuanto a transporte de materiales biológicos potencialmente peligrosos.

Una vez en el laboratorio, la centrifugación y el manejo de la muestra debería de ser similar al procesamiento de muestras de poco volumen, como las pediátricas. Los indicadores habituales de calidad de la muestra, como los índices de hemólisis o lipemia son necesarios, pero probablemente, no suficientes, sobre todo para evaluar la heterogeneidad de la muestra. Para que la muestra de sangre capilar sea de uso habitual en el laboratorio, sería necesario que los autoanalizadores de los laboratorios centrales y reactivos estén adaptados a estos volúmenes tan pequeños de muestra. Uno de los principales problemas es el elevado volumen muerto de los equipos, que suele ser cercano a 100 µL, adaptado a las muestras de sangre venosa. En el caso de usar sangre capilar, unos pocos cientos de microlitros en el mejor de los casos, el volumen muerto debería ser mínimo o idealmente inexistente. Por otra parte, la sangre capilar tiene unas características y composición diferentes de la sangre venosa, ya que es una mezcla de sangre de arteriolas, de los propios capilares y las vénulas. Por lo tanto, las concentraciones de analitos pueden ser diferentes a las encontradas en sangre venosa y se ha de asegurar que los valores de referencia empleados son los correctos y la interpretación clínica es la adecuada. Además, los reactivos, generalmente, están adaptados a sangre venosa y sería necesario realizar comparativas en relación a la sangre capilar. Algunos estudios han mostrado una excelente correlación para algunos analitos, como es el caso de la hemoglobina glicada [4].

Así pues, dadas las grandes potenciales ventajas de las muestras líquidas de sangre capilar respecto a la sangre venosa en relación a la posibilidad de recogida y ahorro de volumen de muestra, su uso, probablemente, se irá incorporando en el laboratorio clínico. Conseguir un buen control de la fase preanalítica, asegurar la trazabilidad de la muestra y validar las metodologías serán retos importantes en este campo para los laboratorios en los próximos años. De ese modo se podrá dar un gran servicio al paciente, al clínico y al sistema, superando ideas de dinosaurios y evitando visiones de unicornios.
